# Management of soil cover and tillage regimes in upland rice-sweet corn systems for better system performance, energy use and carbon footprints

**DOI:** 10.1016/j.heliyon.2024.e26524

**Published:** 2024-02-15

**Authors:** Arindam Kundu, Champak Kumar Kundu, Prithwiraj Dey, Soham Rana, Jhumur Majumder, Anurag Bera, Bappa Paramanik, Partha Sarathi Patra, Md Galal Uddin, Mohamed Abioui, Anuj Saraswat

**Affiliations:** a Department of Agronomy, School of Agriculture and Allied Science, The Neotia University, Sarisha, Jhinga, West Bengal, India; b Department of Agronomy, Bidhan Chandra Krishi Viswavidyalaya, Mohanpur, West Bengal, India; c Agricultural & Food Engineering Department, Indian Institute of Technology Kharagpur, West Bengal, India; d Department of Agronomy, Banaras Hindu University, Varanasi, Uttar Pradesh, India; e Dakshin Dinajpur Krishi Vigyan Kendra, Uttar Banga Krishi Viswavidyalaya, Majhian, West Bengal, India; f Regional Research Station, Terai Zone, Uttar Banga Krishi Viswavidyalaya, Pundibari, Cooch Behar, West Bengal, India; g School of Engineering, University of Galway, Galway, Ireland; h Ryan Institute, University of Galway, Galway, Ireland; i MaREI Research Centre, University of Galway, Galway, Ireland; j Eco-HydroInformatics Research Group (EHIRG), Civil Engineering, University of Galway, Galway, Ireland; k Geosciences, Environment and Geomatics Laboratory (GEG), Department of Earth Sciences, Faculty of Sciences, Ibnou Zohr University, Agadir, Morocco; l MARE-Marine and Environmental Sciences Centre – Sedimentary Geology Group, Department of Earth Sciences, Faculty of Sciences and Technology, University of Coimbra, Coimbra, Portugal; m Department of Soil Science, Govind Ballabh Pant University of Agriculture & Technology, Pantnagar, Uttarakhand, India

**Keywords:** Carbon footprint, Conservation tillage, Live mulching, Global warming potential, Energy use

## Abstract

This study investigates the effects of tillage and mulching regimes on rice-sweet corn systems in the lower Gangetic plains, focusing on region-specific and crop-specific impacts on soil-crop-environmental parameters. The experiment consisted of three levels of tillage: conventional (CT), minimum (MT), and zero (ZT), and four levels of mulching: live, leaf litter, paddy straw, and no mulching. The results show that ZT tillage resulted in higher bulk density (BD) compared to other treatments, despite an increase in soil organic carbon (SOC). Live and leaf litter mulching led to slight reductions in BD in the upper soil layers. CT resulted in net depletion of SOC whereas ZT registered a positive sequestration rate of 1.19 Mg ha^−1^ yr^−1^. Live and leaf litter mulching increased SOC sequestration by 42.6% and 38.8% compared to paddy straw mulching, respectively. Initially, ZT resulted in a 10.3% reduction in system productivity compared to CT, while MT yields were comparable to CT. However, mulching regimes consistently improved production by 16.4%–25.2% as compared to no mulch. ZT and MT were found to be more affordable than CT, with cost savings of 18.2% and 6.8%, respectively. ZT had the highest B: C ratio, indicating better economic efficiency. Among the mulching treatments, live mulching was the most economical. Both ZT and MT saved input energy by approximately 22.9% and 13.5%, respectively compared to CT. Live mulching resulted in the highest net energy and energy output. Compared to CT, ZT reduced carbon footprint (CF) by 41.5 and 22.2% in rice and sweet corn, respectively. MT scored midway between ZT and CT in all parameters. CT exhibited several limitations, including high input energy requirements, high cost of cultivation, poor economic efficiency, negative environmental impacts, and loss of SOC. ZT initially experienced yield reduction and lower net returns in the early years. Therefore, MT was identified as the best alternative in the initial years before transitioning completely to ZT, as it provided comparable yields to CT with better overall benefits. Among the soil cover regimes, live mulching was found to be the most favorable option across all dimensions.

## Highlights

• Conventional tillage depletes soil carbon; zero tillage sequesters 1.19 Mg ha^−1^ yr^−1^.
• Zero tillage cuts productivity by 10.3%; minimum tillage matches conventional.
• Zero and minimum tillage save 18.2% and 6.8% costs; energy saved is 22.9% & 13.5%.
• Zero tillage cuts carbon footprint by 41.5% in rice, 22.2% in sweet-corn.
• Mulching boosts yield 16.4%–25.2% over no mulch.

## Graphical abstract

**Figure:**
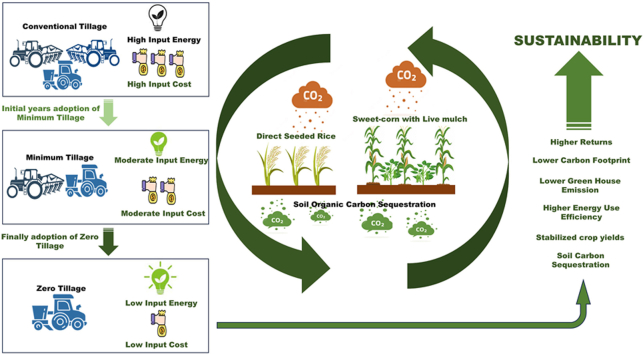

## 1 Introduction

The global emphasis on the economic, environmental, and humane dimensions of sustainable development goals is intensifying and as a foundational pillar of sustainable development, the role of agriculture is indispensable [[1], [2], [3], [4]]. Sustainable agriculture requires careful control of soil cover and soil disturbance since these practices directly impact productivity, soil health, and environmental sustainability [[5], [6], [7]]. Rice is the main crop crucial for food security and rural livelihoods in the lower Gangetic Plains, a sizable portion of India's Indo-Gangetic Plains [8,9]. Labour availability for rice transplanting has been there as a persistent problem and increasing wages and decreasing the timely availability of labourers can be a major driving force for the gradual shift to direct seeded rice (DSR) in the region. Wintersweet corn has been a recent addition to the cropping system, especially in the peri-urban area due to its huge economic potential and profitability in recent years. Several management elements, such as soil management techniques like tillage and soil cover, can impact the productivity and sustainability of rice-sweet corn systems in this region. The impact of various tillage regimes on crop performance and soil characteristics in diverse agricultural systems worldwide has been the subject of numerous researches [[10], [11], [12], [13]]. Traditional methods like conventional tillage (CT), which include heavy mechanical soil disturbance via ploughing and harrowing, have been identified as contributing factors to soil deterioration, including decreased soil organic carbon (SOC) content, increased soil erosion, and lower fertility [[14], [15], [16], [17], [18]]. Conservation tillage techniques like zero tillage (ZT), reduced tillage, and minimum tillage (MT) have arisen as viable substitutes. While seeds are directly sown into untilled soil in ZT, much-reduced soil disturbance is ensured using reduced tillage tools in MT. Reduced soil erosion, improved water infiltration and retention, higher SOC content, and improved soil structure are just a few potential advantages that these techniques can provide in the long run [[19], [20], [21], [22], [23]]. Additionally, conservation tillage techniques have the potential to maintain soil fertility, soil productivity, decrease labour requirements, increase profitability, energy efficiency, and reduce greenhouse gas (GHG) emissions [[24], [25], [26], [27]].

Mulching or maintaining a soil cover though seems to be a very simplistic technique but can be a game changer in influencing crop performance and soil health largely [[28], [29], [30], [31]]. Mulching helps regulate soil temperature, conserve soil moisture, suppress weed growth, enhance nutrient cycling, improve soil structure and enzymatic activity. Different mulching materials, such as paddy straw, leaf litter, etc. have been used in various cropping systems to examine their effects on soil properties and crop yields [30]. The addition of a legume crop as live mulch to the system can have a dual positive impact on the system from the nitrogen fixation point of view and also provide economic yield in some cases. Despite this, mulching is not always beneficial. Mulching can reduce the amount of solar radiation that reaches the soil surface, thereby delaying crop growth and development by lowering soil temperatures [32]. Therefore, it is important to add an adequate amount of mulch to prevent adverse effects on early seedling growth, water use efficiency, and crop yield, since straw decomposes in soil and competes with crops for nitrogen [[33], [34], [35]].

While previous studies have addressed the influence of tillage regimes and mulching on crop performance and soil parameters, the specific interplay of these factors within rice-sweet corn systems of the lower Gangetic Plains remains poorly covered. Most of the studies in this region are concentrated on the upper Gangetic Plains and Middle Gangetic Plains specifically with the rice-wheat system. This knowledge gap is not trivial: studies confirm that the implications of these management practices exhibit region-specific and crop-specific variations [36,37]. Soil carbon has been accepted as a central element to the soil health concept and it is dictated by the management-soil-plant-climatic interactions which show spatio-temporal influences. Dependencies of soil carbon built-up and resulting soil physical-chemical-biological parameters show significant regional and crop-specific impacts. The unique agro-climatic conditions, soil characteristics, and agricultural practices in the lower Gangetic Plains necessitate a tailored evaluation for optimizing crop yield, energy efficiency, and sustainability. Moreover, while individual effects of tillage or mulching have been explored, the combined and synergistic impact of these, especially with the inclusion of a live mulching system within a rice-sweet corn framework, remains an under-researched area. Thus, to formulate holistic soil management approaches that enhance productivity while mitigating environmental impacts, it's imperative to decode the multifaceted interactions of these practices. It's against this backdrop that our study introduces a pioneering exploration of the combined effects of different tillage and mulching strategies, specifically within the rice-sweet corn systems of the lower Gangetic Plains.

The core objective of this research is to delve deeply into alterations in soil physicochemical properties over time, emphasizing metrics like bulk density (BD), SOC content, and the long-term trajectory of SOC sequestration. Beyond this, the study aspires to quantify the potential of SOC sequestration, to discern the balance of energy inflow, outflow, and net yield associated with diverse soil management practices, and to compute the carbon footprint (CF) of each, revealing their contributions to global warming. The impetus for this comprehensive investigation stems from a recognized knowledge gap in the domain; our results aim to provide a robust understanding of the interactive and combined effects of different soil management methods. The implications of our findings transcend mere academic interest. For on-the-ground actors like farmers, policy architects, and other agricultural stakeholders, this study's outcomes could facilitate the shift towards environmentally sound practices that concurrently enhance yield, optimize energy use, and mitigate ecological harm, thus paving the path to a truly sustainable agricultural future.

## 2 Materials and methods

### 2.1 Experimental site

**Fig. 1:**
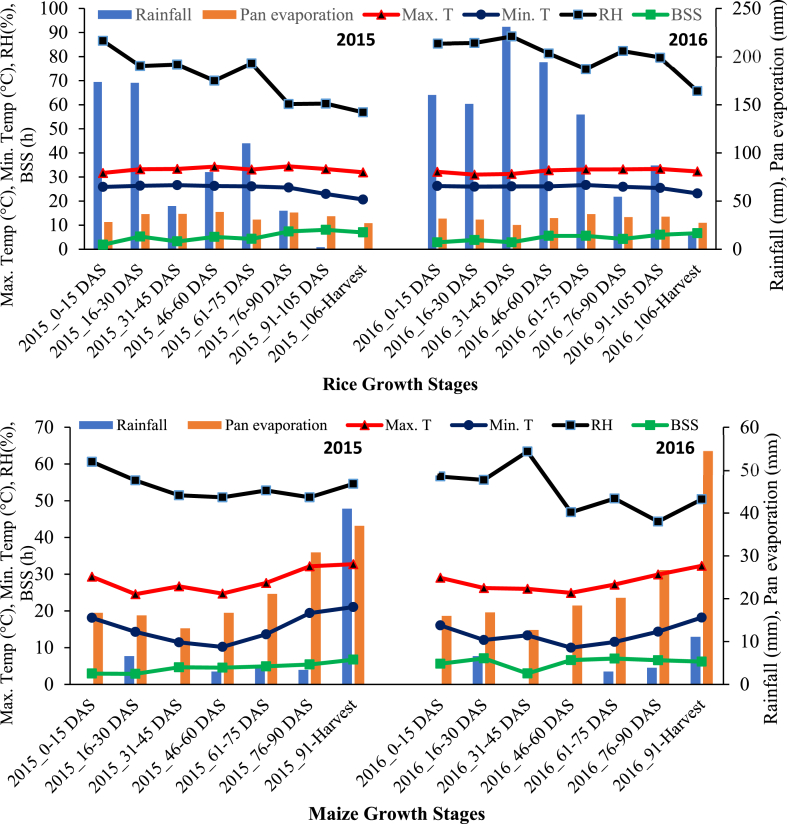
Prevailing weather parameters during rice and sweet corn growth stages during 2015–2017. Note: ‘Min. T.’ stands for Minimum Temperature; ‘Max. T.’ stands for Maximum Temperature; ‘RH’ denotes Relative Humidity; ‘Pan Evap.’ denotes Pan Evaporation; ‘BSS’ stands for Bright Sunshine Hours; ‘DAS’ stands for Days After Sowing.

The current experiment was conducted from 2015 to 2019 at the Central Research Farm of Bidhan Chandra Krishi Viswavidyalaya, West Bengal, India (22°58 N, 88°31 E, 9.75 m above mean sea level). The experimental site enjoys a tropical sub-humid climatic zone of Eastern India with an average annual rainfall of 1608 mm. The prevalent weather conditions during the experimental period are depicted in Fig. 1. The details of the initial soil condition at the experimental site are furnished in Table 1. In the current study soil parameters like BD, SOC, amount of SOC sequestration, and SOC sequestration rate were estimated. The soil samples were collected using a core sampler for the estimation of BD from 0 to 15, 15–30, 30–45, and 45–60 cm depth. For SOC estimation, composite soil samples were collected from 0 to 15, 15–30, 30–45, and 45–60 cm depth with the help of an auger, and SOC was estimated using the Walkley and Black method.

**Table 1:** Initial soil parameters of experimental site.

Parameters	Values	Methods
Textural class		Hydrometer method [38]
Sand (%)	60.19	
Silt (%)	20.73	
Clay (%)	19.08	
Bulk density (Mg m^−3^)	1.49	Core sampler [39]
pH (1:2.5 soil:water)	7.2	pH meter [40]
Organic carbon (Mg ha^−1^)	21.89	Walkley and Black method [40]
Available N (kg ha^−1^)	176.39	Alkaline KMnO_4_ method [40]
Available P (kg ha^−1^)	33.0	Olsen method [41]
Available K (kg ha^−1^)	150.61	Neutral 1 N NH_4_OAc method [41]

### 2.2 Experimental details

Before the start of the current experiment, initially, fallow land was initially brought into cultivation in 2012 with a cropping sequence of rice-oat for the initial year and sweet corn-berseem-summer mung for subsequent years. The current experiment was initiated from Kharif (rainy) 2015 with direct seeding of upland rice variety IET 17509. In the Rabi (winter) season, the hybrid sweet corn variety VMH-125 was grown in succession. Rice was sown in the second week of July and harvested in the second week of November. At the same time, sweet corn was planted in the last week of November and harvested in the second week of March. Row-to-row spacing's for rice and sweet corn were 25 cm and 50 cm, respectively, with a seed rate of 30 kg ha^−1^ for both the crops and a 15 cm plant-to-plant spacing was maintained for Rabi sweet corn, whereas rice was sown as continuous rows with dry direct seeding. Rice was grown in rainfed conditions while succeeding Rabi sweet corn was irrigated with 4–5 irrigations.

The experiment was conducted in a split plot design with three main plot treatments and four sub-plot treatments, replicated thrice. Three tillage regimes were assigned to the main plots: CT, ZT, and MT. The sub-plot treatments consisted of four mulching regimes: live mulching, paddy straw mulching, leaf litter mulching, and no mulching. The CT plots were cultivated twice with a tractor-drawn cultivator and single cross-operation of a rotavator, respectively, followed by levelling. The MT plots were cultivated once with a tractor-drawn cultivator. While no-tillage operation except limited secondary tillage at the time of sowing done in ZT plots. Similar land preparation methods were adopted for Rabi sweet-corn also. Glyphosate 41% SL was sprayed in the fallow land at 2.5 L ha^−1^, ten days before sowing. All four levels of mulching were applied in the Rabi season only. For live mulching, a row of cowpeas was sown in furrows (manually opened) between every two rows of sweet corn at 10 kg ha^−1^. Paddy straw mulch was applied at 4 t ha^−1^(dry weight) basis and leaf litter mulching was carried out by broadcasting dried leaves, twigs, and barks of locally available trees (*Lagerstroemia speciosa* and *Stereospermum chelonoides*) between the rows of sweet-corn at a 3 t ha^−1^ basis.

Recommended doses of nitrogen (N), phosphorous (P_2_O_5_), and potassium (K_2_O) for rice and sweet corn were 60:30:30 and 100:50:50 kg ha^−1^, respectively. Half of N and a complete dosage of P_2_O_5_ and K_2_O were applied at final land preparation (with zero-till drill in case of ZT plots). The remaining 50% of N was top dressed during maximum tillering and panicle initiation stages, respectively, in two equal splits for rice. Similar top dressings were applied for the second and third irrigation in the case of sweet corn. The system productivity (t ha^−1^) was calculated in terms of Rice Equivalent Yield (REY) using Eq. (1):

(1) $$\text{REY}\left(t\;{\text{ha}}^{-1}\right)=\frac{\text{Sweet}\;\text{corn}\;\text{yield}\;\left(t\;{\text{ha}}^{-1}\right)\times \text{Price}\;\text{of}\;\text{Sweet}\;\text{Corn}\;\left(\text{USD}\;t^{-1}\right)}{\text{Price}\;\text{of}\;\text{Rice}\;\left(\text{USD}\;t^{-1}\right)}+\text{Rice}\;\text{yield}\left(t\;{\text{ha}}^{-1}\right)$$

The price of rice was taken as the minimum support price declared by Govt. of India for the respective years. The price of sweet corn was determined by the prevailing local market prices. The average INR to USD conversion factors of the respective years were used for all the economic calculations. The gross return (GR) was calculated by multiplying individual crop yields with their prices and summing up. Net return (NR) was calculated by subtracting the cost of cultivation (CoC) from GR. The B: C ratio was calculated by dividing GR with CoC.

### 2.3 Energy parameters, carbon footprint and GWP

The system energy parameters, such as total energy inputs (Eq. (2)) and total energy outputs (Eq. (3)) at a system level, were calculated using energy equivalents suggested by different literature shown in Table 2. The other system energy parameters were calculated using the following formula (Eqs. (4)–(7)):

(2) *Total Energy Input (GJ ha*^*−*^^*1*^*) = Energy Input in Rice (GJ ha*^*−*^^*1*^*) + Energy Input in Sweet Corn (GJ ha*^*−*^^*1*^*)*

(3) *Total Energy Output (GJ ha*^*−*^^*1*^*) = Energy Output in Rice (GJ ha*^*−*^^*1*^*) + Energy Output in Sweet Corn (GJ ha*^*−*^^*1*^*)*

(4) *Net System Energy (GJ ha*^*−*^^*1*^*) = Total energy output (GJ ha*^*−*^^*1*^*) – Total energy input (GJ ha*^*−*^^*1*^*)*

(5) $$System\;Energy\;Profitability\;\left(t\;{\mathrm{ha}}^{-1}\right)=\frac{Net\;Energy\;\left(MJ\;{ha}^{-1}\right)}{Total\;Energy\;Input\;\left(MJ\;{ha}^{-1}\right)}$$

(6) $$System\;Specific\;Energy\left(MJ\;kg^{-1}\right)=\frac{Energy\;input\;\left(MJ\;{ha}^{-1}\right)}{Rice\;equivalent\;yield\;\left(kg\;{ha}^{-1}\right)}$$

(7) $$System\;Energy\;Productivity\left(g\;MJ^{-1}\right)=\frac{Rice\;equivalent\;yield\;\left(g\;{ha}^{-1}\right)}{Total\;Energy\;Input\;\left(MJ\;{ha}^{-1}\right)}$$

**Table 2:** Energy equivalents and CO_2_ equivalents of different inputs and outputs of rice-sweet corn system.

Particulars	Energy Equivalent (MJ unit^−1^)	CO_2_ equivalent (kg CO_2_ eq. unit^−1^)	References
Human labourer	1.96 MJ h^−1^	0.86 kg manday^−1^	[42,43]
Diesel	56.31 MJ L^−1^	3.32 kg L^−1^	[42,43]
Tractor	68.40 MJ kg^−1^	3.32 kg h^−1^	[42,44]
Farm machinery	62.10 MJ kg^−1^	3.32 kg h^−1^	[45,38]
Pump	62.70 MJ kg^−1^	3.32 kg h^−1^	[42,46]
Combine harvester	116.00 MJ kg^−1^	3.32 kg h^−1^	[42,46]
Rice seed	14.70 MJ kg^−1^	5.1 kg kg^−1^	[43,47]
Corn seed	14.57 MJ kg^−1^	5.1 kg kg^−1^	[43,48]
Cowpea seed	14.70 MJ kg^−1^	5.1 kg kg^−1^	[43,49]
Leaf litter mulch	14.70 MJ kg^−1^	2.78 kg kg^−1^	[47]
Water	1.02 MJ m^−3^	–	[43]
Nitrogen	66.14 MJ kg^−1^	4.96 kg kg^−1^	[43]
Phosphorous (P_2_O_5_)	12.44 MJ kg^−1^	1.35 kg kg^−1^	[43]
Potassium (K_2_O)	11.15 MJ kg^−1^	0.58 kg kg^−1^	[43]
Fungicides	92.00 MJ kg^−1^	3.9 kg L^−1^	[42,50]
Herbicides	238.00 MJ kg^−1^	6.3 kg L^−1^	[42,51]
Insecticides	199.00 MJ kg^−1^	5.1 kg L^−1^	[42,50]
Paddy (raw)	14.70 MJ kg^−1^	–	[52]
Paddy straw	12.50 MJ kg^−1^	–	[52]
Paddy straw mulch	10.00 MJ kg^−1^	0.01 kg kg^−1^	[47,52]
Corn (green cob)	17.00 MJ kg^−1^	–	[53]
Corn stover	12.50 MJ kg^−1^	–	[53]
Cowpea biomass	18.00 MJ kg^−1^	–	[49]

The environmental impacts of tillage and mulches were estimated using yield-scaled CF and Global Warming Potential (GWP). The GWP was calculated as the total amount of CO_2_ and N_2_O emissions during crop production in terms of CO_2_ equivalents (Yadav et al., 2018). Emission of CH_4_ from direct seeded upland rice was not considered due to its negligible amount. The CO_2_ equivalent of N_2_O was calculated using a factor of 265 suggested by IPCC (2013). The calculations of greenhouse emissions and emission coefficients in Table 2 were used. The N_2_O emission from applied fertilizers, manure, and crop residue was calculated using Eq. (8) [54].

(8) $$N_{2}O\;emissions\;\left(kg\;year^{-1}\right)\;=N\;\left(kg\;year^{-1}\right)\times \;EF\;\times \frac{44}{28}$$

Where, N is the yearly nitrogen input through fertilizers, manures, residues, etc., and EF is emission factor for N_2_O (kg N_2_O/kg N input). The value of EF was taken as 0.01. The GWP was calculated using Eq. (9).

(9) *GWP (kg year*^*−1*^*) = CO*_*2*_*Emission + (N*_*2*_*O Emission × 265)*

The yield-scaled CFs for component crops was calculated using the method described by Ref. [55] with improvisation for componential calculations using Eqs. (10), (11)):

(10) $$CF_{Rice}\left(kg\;CO_{2}\;equivalent\;t^{-1}\right)=\frac{GWP\;of\;Rice}{Rice\;yield}$$

(11) $$CF_{Sweet\;corn}\;\left(kg\;CO_{2}\;equivalent\;t^{-1}\right)\;=\frac{GWP\;of\;Sweet\;Corn}{Rice\;Equivalent\;Yield\;of\;sweet\;corn}$$

### 2.4 Statistical analysis

The data from the current experiment were subjected to the multi-year combined analysis of variance (ANOVA) in a split-plot arrangement for four cropping cycles (pooled) as described by Ref. [56]. Mean separation of the data after conducting ANOVA was done using Fisher's least significant difference (LSD) method at p < 0.05. Treatments bearing no common letters followed by the table values in superscript indicate statistically significant differences between them at p < 0.05. All the statistical analysis has been done with the help of SAS [57] and SPSS v.25 [58].

## 3 Results

### 3.1 Effect of tillage and mulching regimes on soil bulk density

**Fig. 2:**
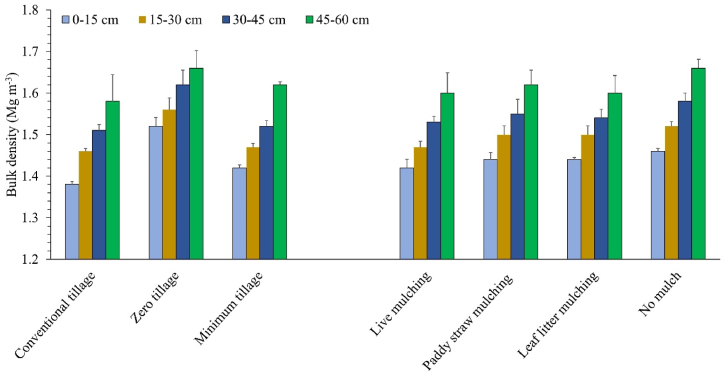
Effect of different tillage and mulching regimes on soil bulk density.

Soil BD increased with the increasing soil depth across the tillage and mulching treatments (Fig. 2). Under ZT, BD was consistently higher than CT plots throughout four soil depths (0–15, 15–30, 30–45, and 45–60 cm).In the 0–15 cm soil layer, ZT recorded the highest BD, which was 10.1% higher than BD recorded in CT plots; MT plots had been registered with intermediate BD, which was 6.6% lesser than ZT plots. In deeper soil depths of 15–60 cm, minimum and conventional tillage resulted in statistically similar BDs. The difference in BD between ZT and CT plots narrowed to only 4.8% at deeper depths. The current experiment has recorded no effect of any mulching regime on soil BD at different depths except 0–15 cm layer where live mulching resulted in significantly lower BD than other treatments. The interaction effects of tillage and mulching practices were non-significant across all the soil depths.

### 3.2 Soil organic carbon sequestration and sequestration rate

Significant effect of different tillage practices and mulching on SOC after two complete cycles of the rice-sweet corn system (Table 3). The plots under ZT recorded a significantly higher SOC content (24.27 Mg ha^−1^) and rate of carbon sequestration (1.19 Mg ha^−1^ yr^−1^) compared to MT and CT. The CT resulted in a net loss of SOC (−0.76 Mg ha^−1^) over the years, while the ZT resulted in a significant build-up of SOC resulting in sequestration. The MT plots somewhat maintained the SOC without any significant positive or negative sequestration. Although both the ZT and MT plots initially had the same amount of organic carbon (21.89 Mg ha^−1^), the ZT plots showed 10.7% higher SOC at the end of the experiment. The ZT plots have also been recorded with the highest net positive sequestration rate of 1.19 Mg ha^−1^ yr^−1^, while CT plots have shown a negative rate of SOC sequestration.

**Table 3:** Effect of tillage and mulching regimes on soil organic carbon sequestration rate after two complete cycles of rice-sweet corn system.

Treatments	Final soil organic carbon (Mg ha^−1^)	Soil organic carbon sequestered (Mg ha^−1^)	Sequestration rate (Mg ha^−1^ yr^−1^)
*Tillage regimes (T)*			
Conventional tillage	21.13 ± 0.14^b^	−0.76 ± 0.03^c^	−0.38 ± 0.07^c^
Zero tillage	24.27 ± 0.23^a^	2.39 ± 0.12^a^	1.19 ± 0.43^a^
Minimum tillage	21.93 ± 0.13^b^	0.04 ± 0.01^b^	0.02 ± 0.003^b^
*Mulching regimes (M)*			
Live mulching	22.66 ± 0.16^a^	0.77 ± 0.11^a^	0.39 ± 0.09^a^
Paddy straw mulching	22.43 ± 0.21^b^	0.54 ± 0.18^b^	0.27 ± 0.10^b^
Leaf litter mulching	22.64 ± 0.34^a^	0.75 ± 0.12^a^	0.38 ± 0.11^a^
No mulch	22.04 ± 0.28^c^	0.18 ± 0.02^c^	0.18 ± 0.02^c^
**Interaction (TxM)**	ns	ns	ns
**Initial soil organic carbon (Mg ha**^**−**^**^1^)**	**21.89**		

Note: Treatments carrying common letters are statistically on par while significant difference at p = 0.05 between the treatment mean is indicated by presence of no common letters between the treatments under comparison; ‘ns’ stands for non-significant at p = 0.05.

Among the different mulching regimes, live mulching (22.66 Mg ha^−1^) and leaf litter mulching (22.64 Mg ha^−1^) had the most significant impact on soil carbon storage and carbon sequestration rate and were statistically on par. On the other hand, paddy straw mulching and no mulch treatment recorded lower final SOC. The amount of organic carbon sequestered has also shown similar trends. Compared with no mulch treatments, live mulching, leaf litter mulching, and paddy straw mulching resulted in 116.750.0 and 111.1% increases in SOC sequestration rate, respectively. All the mulching treatments resulted in favorable sequestration rates, and live mulching and leaf litter mulching were recorded with the highest sequestration rates, followed by paddy straw mulching. The interaction effect between tillage and mulching was non-significant on SOC sequestration.

### 3.3 Yield and system productivity

The tillage system significantly impacted the yield of rice, sweet corn, and the system yield. Plots under CT and MT were recorded with statistically on-par results. In contrast, a significant yield decrease of 15.1% was recorded under ZT concerning CT plots (Table 4). Similarly, under CT, the highest grain yield of sweet corn (12.78 t ha^−1^) was recorded, which was on par with MT but invariably resulted in a significant yield decrease of 11.7% in ZT. System yield in terms of REY also showed a similar pattern, with MT and CT achieving statistical parity and resulting in significantly higher yields than ZT. Among the mulching treatments, live mulching recorded the highest rice yield (3.36 t ha^−1^) but was statistically similar to yields from other mulching treatments except for no mulch plots. Similarly, the highest sweet-corn yield was recorded under live mulching, which was statistically similar to leaf litter mulched plots. Rice-sweet corn system yield in terms of REY was also significantly higher under live mulching with legumes, which was statistically on par with leaf litter mulching. For non-mulched plots, mulching increased the REY to the tune of 25.2, 16.5, and 23.4% with live mulch, paddy straw mulch, and leaf litter mulch, respectively. Interaction effects of tillage and mulching on rice, sweet-corn yield, and REY were found significant. A combination of CT with live mulching invariably resulted in the highest yields. In all the tillage regimes, live mulching had resulted in increased yield.

**Table 4:** Effect of tillage and mulching regimes on rice yield, sweet corn yield and system yield after two complete cycles of rice-sweet corn system.

Treatments	Live mulching	Paddy straw mulching	Leaf litter mulching	No mulch	Mean
*Rice yield (t ha*^*−*^*^1^)*					
Conventional tillage	3.92 ± 0.52	3.20 ± 0.28	3.49 ± 0.34	2.86 ± 0.20	***3.37 ± 0.33***^***a***^
Zero tillage	3.02 ± 0.37	2.89 ± 0.25	2.95 ± 0.28	2.60 ± 0.10	***2.86 ± 0.25***^***b***^
Minimum tillage	3.16 ± 0.34	3.06 ± 0.31	3.10 ± 0.30	2.84 ± 0.18	***3.04 ± 0.28***^***a***^
**Mean**	***3.36 ± 0.41***^***a***^	***3.05 ± 0.28***^***ab***^	***3.18 ± 0.30***^***a***^	***2.77 ± 0.16***^***b***^	
*Sweet corn yield (t ha*^*−*^*^1^)*					
Conventional tillage	13.91 ± 2.25	12.55 ± 2.01	13.40 ± 1.70	11.25 ± 1.34	***12.78 ± 1.80***^***a***^
Zero tillage	12.02 ± 1.70	11.39 ± 1.34	11.56 ± 1.18	10.21 ± 0.91	***11.29 ± 1.28***^***b***^
Minimum tillage	13.08 ± 2.78	12.09 ± 1.64	12.77 ± 2.43	10.49 ± 1.06	***12.11 ± 1.97***^***ab***^
**Mean**	***13.00 ± 2.25***^***a***^	***12.01 ± 1.63***^***b***^	***12.58 ± 1.77***^***ab***^	***10.65 ± 1.11***^***c***^	
*System yield (REY t ha*^*−*^*^1^)*					
Conventional tillage	33.61 ± 6.11	32.64 ± 6.77	34.48 ± 5.22	29.15 ± 4.43	***32.37 ± 5.70***^***a***^
Zero tillage	31.40 ± 5.75	29.23 ± 4.28	29.77 ± 3.32	25.8 ± 3.76	***29.03 ± 4.12***^***b***^
Minimum tillage	35.75 ± 10.23	31.51 ± 5.35	34.75 ± 9.03	25.55 ± 3.53	***31.82 ± 6.92***^***a***^
**Mean**	***33.46 ± 7.01***^***a***^	***31.15 ± 5.45***^***b***^	***32.99 ± 5.81***^***ab***^	***26.73 ± 4.09***^***c***^	

Note: Treatments carrying common letters are statistically on par while significant difference at p = 0.05 between the treatment mean is indicated by presence of no common letters between the treatments under comparison; ‘ns’ stands for non-significant at p = 0.05.

### 3.4 Production economics

The system's economic efficiency has been calculated after factoring in all the costs associated with the different packages and practices. The CoC was highest (512 USD ha^−1^) in CT plots, which was 22.1 and 7.3% higher than the cost incurred in ZT and MT plots. The highest NR (982 USD ha^−1^) was recorded from CT plots which were on par with MT plots. The ZT plots resulted in the lowest NR, but it was statistically similar to the returns achieved in MT. In terms of the B: C ratio, the ZT plots resulted in a significantly higher B: C ratio than the other tillage regimes. The CT and MT plots resulted in a statistically similar B: C ratio. Among the mulching regimes, paddy straw mulching incurred the highest CoC. Live mulching closely followed paddy straw mulching in terms of cost involvement. However, live mulching with legumes resulted in the highest NR, 41.1% higher than no mulch treatment. It was followed by leaf litter mulching and paddy straw mulching. The highest B: C ratio was recorded from live mulching, which was statistically similar to leaf litter mulching. No mulch treatments resulted in the lowest B: C ratio (Table 5).

**Table 5:** Effect of tillage and mulching regime on economic performance of rice-sweet corn system.

Treatments	Cost of cultivation (USD ha^−1^)	Net return (USD ha^−1^)	B:C
*Tillage regimes (T)*			
Conventional tillage	512	982 ± 28^a^	2.91 ± 0.09^b^
Zero tillage	419	860 ± 45^b^	3.05 ± 0.07^a^
Minimum tillage	477	920 ± 33^a^	2.92 ± 0.10^b^
*Mulching regimes (M)*			
Live mulching	480	1080 ± 57^a^	3.25 ± 0.11^a^
Paddy straw mulching	487	902 ± 39^b^	2.85 ± 0.13^b^
Leaf litter mulching	473	983 ± 42^ab^	3.07 ± 0.09^a^
No mulch	467	765 ± 23^c^	2.63 ± 0.11^c^
**Interaction (TxM)**	–	s	ns

Note: Treatments carrying common letters are statistically on par while significant difference at p = 0.05 between the treatment mean is indicated by presence of no common letters between the treatments under comparison; ‘ns’ stands for non-significant at p = 0.05 and ‘s’ denotes significance of the interaction effects. ‘B:C’ stands for Benefit-Cost Ratio.

### 3.5 System energetics

The tillage system significantly impacted total input, output, and net energy. It was recorded that CT plots had the highest input and output energy, followed by MT and ZT. A significant decrease of 22.9% of input energy was recorded under ZT treatment and a 13.5% decrease under MT to CT plots (Table 6). Similarly, the highest total output energy was recorded under CT plots, which was statistically similar to MT plots. The ZT plots have performed equally with the MT plots, resulting in only a slight decrease in the total output energy of 6.3%. Net energy showed a resemblance with trends shown by output energy. The highest net output energy was recorded in CT plots which were at par with MT plots. Though ZT plots have resulted in the lowest net energy it was only 5.6% less than the MT plots. Mulching has shown a significant effect on energy balance. Plots with Paddy straw mulching had shown the highest total input energy (27.5 GJ ha^−1^) compared to leaf litter mulching (25 GJ ha^−1^) and live mulching (22.1 GJ ha^−1^) and no mulching (21.6 GJ ha^−1^). Live mulched plots showed the highest net energy which was 6.4% higher than leaf litter mulched and 13.5% higher than paddy straw mulched plots. Total output energy showed identical results with net energy. Live mulched plots resulted on par with leaf litter mulched plots and 8.5% higher net energy than paddy straw mulched plots and 22.6% higher net energy than no mulched plots.

**Table 6:** Effect of tillage and mulching regimes on total input, output, and net energy of rice-sweet corn system.

Treatments	Live mulching	Paddy straw mulching	Leaf litter mulching	No mulch	Mean
*Total input energy (GJ ha*^*−*^*^1^)*					
Conventional tillage	25.4 ± 0.5	30.9 ± 0.3	28.4 ± 0.6	25.0 ± 0.6	**27.4 ± 0.3**^**a**^
Zero tillage	19.1 ± 0.3	24.6 ± 0.6	22.1 ± 1.1	18.7 ± 0.2	**21.1 ± 0.3**^**c**^
Minimum tillage	21.7 ± 0.5	27.1 ± 0.4	24.6 ± 0.8	21.3 ± 0.3	**23.7 ± 0.4**^**b**^
**Mean**	**22.1 ± 0.7**^**b**^	**27.5 ± 0.2**^**a**^	**25.0 ± 1.1**^**a**^	**21.6 ± 0.5**^**b**^	
*Total output energy (GJ ha*^*−*^*^1^)*					
Conventional tillage	221.2 ± 12.3	194.1 ± 17.1	206.9 ± 16.5	166.9 ± 16.9	**197.3 ± 14.3**^**a**^
Zero tillage	181.5 ± 9.9	171.2 ± 15.3	176.7 ± 16.9	153.8 ± 10.1	**170.8 ± 12.9**^**b**^
Minimum tillage	192.5 ± 5.8	183.1 ± 9.1	188.6 ± 9.9	164.5 ± 8.6	**182.2 ± 10.1**^**ab**^
**Mean**	**198.4 ± 6.9**^**a**^	**182.8 ± 14.9**^**b**^	**190.7 ± 13.0**^**ab**^	**161.8 ± 12.1**^**c**^	
*Net energy (GJ ha*^*−*^*^1^)*					
Conventional tillage	195.8 ± 6.9	163.2 ± 12.5	178.5 ± 8.6	141.9 ± 13.1	**169.9 ± 12.1**^**a**^
Zero tillage	162.4 ± 5.4	146.6 ± 8.9	154.6 ± 10.2	135.1 ± 7.7	**149.7 ± 9.8**^**b**^
Minimum tillage	170.9 ± 7.0	155.9 ± 12.1	164.0 ± 8.9	143.3 ± 8.9	**158.5 ± 10.9**^**ab**^
**Mean**	**176.4 ± 16.8**^**a**^	**155.3 ± 10.1**^**b**^	**165.7 ± 9.1**^**ab**^	**140.1 ± 9.8**^**c**^	

Note: Treatments carrying common letters are statistically on par while significant difference at p = 0.05 between the treatment mean is indicated by presence of no common letters between the treatments under comparison; ‘ns’ stands for non-significant at p = 0.05.

The specific energy of CT plots was highest (846.4 MJ ha^−1^) as compared to MT (744.8 MJ ha^−1^) and ZT (726.8 MJ ha^−1^) plots. The specific energy of ZT was similar to MT plots. Among mulching regimes, no mulched plots showed 9.77% lower specific energy than paddy straw mulching and 6.22% greater than leaf litter mulched plots. Zero-tilled plots and minimum-tilled plots showed insignificant differences in system energy productivity. Conventionally tilled plots showed 14.1% lower system energy productivity than zero tillage and 12.1% lesser than minimum tillage. Live mulched plots showed the highest system energy productivity, followed by leaf litter mulching, no mulching, and paddy straw mulching. Paddy straw mulching showed 14.5% lesser system energy productivity than leaf litter mulching and 8.91% less than no mulching plots. System energy profitability of plots with zero tillage showed statistically at per result with minimum tillage and 14.51% greater than conventional tilled plots. Live mulching had shown the highest system energy profitability (7.9 MJ MJ^−1^). Leaf litter mulched plots showed only 1.5% greater system energy profitability than no mulched plots and 17.8% greater than paddy straw mulched plots (Table 7).

**Table 7:** Effect of tillage and mulching regimes on specific energy, system energy productivity and system energy profitability of rice-sweet corn system.

Treatments	Specific energy (MJ kg^−1^)	System Energy productivity (g MJ^−1^)	System Energy profitability (MJ MJ^−1^)
*Tillage regimes (T)*			
Conventional tillage	846.4 ± 51.3^a^	1181.3 ± 56.9^b^	6.2 ± 0.8^b^
Zero tillage	726.8 ± 67.8^b^	1375.8 ± 55.7^a^	7.1 ± 0.7^a^
Minimum tillage	744.8 ± 44.3^b^	1342.6 ± 67.1^a^	6.7 ± 0.7^ab^
*Mulching regimes (M)*			
Live mulching	648.5 ± 23.0^c^	1541.9 ± 34.9^a^	7.9 ± 1.1^a^
Paddy straw mulching	887.1 ± 54.7^a^	1127.2 ± 67.1^d^	5.6 ± 0.8^c^
Leaf litter mulching	757.8 ± 66.4^b^	1319.6 ± 54.8^b^	6.6 ± 0.5^ab^
No mulch	808.1 ± 75.1^ab^	1237.5 ± 56.8^c^	6.5 ± 0.4^b^
**Interaction (TxM)**	s	s	s

Note: Treatments carrying common letters are statistically on par while significant difference at p = 0.05 between the treatment mean is indicated by presence of no common letters between the treatments under comparison; ‘s’ stands for significance of interaction effects at p = 0.05.

### 3.6 System carbon footprints and global warming potentials

**Fig. 3:**
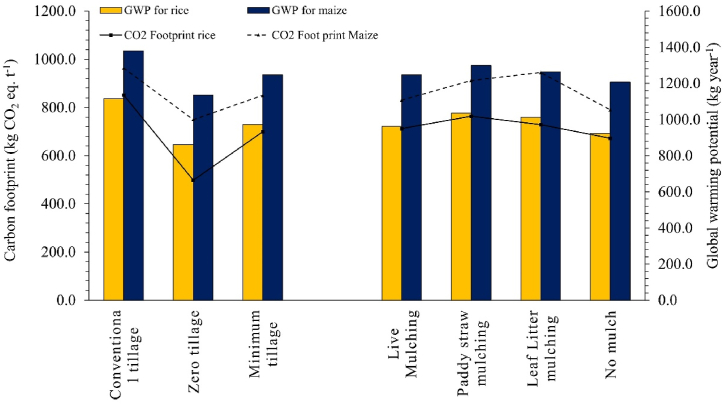
Effect of different tillage and mulching regimes on carbon footprints and global warming potentials of rice-sweet corn system.

The CFs and GWPs of rice-sweet corn cropping systems significantly depended upon different mulching practices and tillage regimes. The CT plots have shown the highest CF in rice (851.4 kg eq. CO_2_ ha^−1^) and sweet corn (962.9 kg eq. CO_2_ ha^−1^). The CF of ZT plots for rice was 70.8% less than CT plots and 28.75% less than MT. Paddy straw mulched plots had shown statistically on per in CF in rice fields with leaf litter mulching (Fig. 3). No mulched plots had shown minimum emission of CO_2_ in rice fields, which is also 5.6% lesser than live mulched plots and 7.7% lesser than leaf litter mulched plots. In the same line, GWPs for rice (1114.4 kg year^−1^) and sweet corn (1379.3 kg year^−1^) were highest in CT plots. The ZT plots have been observed with the lowest GWPs (861.4 kg year^−1^ for rice and 1135.9 kg year^−1^ for sweet corn). Paddy straw mulched plots had shown the highest global warming potential, followed by leaf litter and live mulching. In Table 8, a relative comparison of energetics and carbon footprints have been made from similar studies with Rice-Maize systems. The energy input ranged from 24.5 to 105.2 GJ ha^−1^ for conventional tillage. While for conservation tillage, on an average input energy varied from 21.1 to 93.8 GJ ha^−1^. In a similar way retention of mulch or residue resulted in varied energy input from 18.5 to 133.7 GJ ha^−1^. A wide variation in the output energy, net energy as well as carbon foot print was evident from similar studies.

**Table 8:** Comparison of energetics and carbon foot prints from similar studies conducted in different locations.

Treatments	Energy Input (GJ ha^−1^)	Energy Output (GJ ha^−1^)	Net Energy (GJ ha^−1^)	Carbon foot print (kg CO_2_ eq. t^−1^)	Reference
CT	27.4	197.3	169.9	1812.9	This study
CST	21.1	170.8	149.7	1247.5	
M/R	27.5	182.8	155.3	1675.8	
NM	21.6	161.8	140.1	1463.1	
CT	24.5	206.9	182.4	2071.0	[43]
CST	18.3	183.1	164.8	1648.7	
M/R	18.5	182.8	164.4	1739.0	
NM	18.1	179.3	161.3	1549.5	
CT	64.9	179.1	114.1	630	[59]
CST	52.4	170.0	117.5	235	
M/R	64.9	195.1	130.1	149	
NM	51.9	175.0	123.2	214	
CT	105.2	163.3	58.1	–	[60]
CST	93.8	168.9	75.1	–	
M/R	133.7	170.1	36.4	–	
NM	62.1	157.9	95.8	–	

Note: CT: Conventional tillage; CST: Conservation tillage; M/R: With mulching/residue; NM: No mulching/residue.

## 4 Discussion

Soil BD is inherently predisposed to rise with increased soil depth. This phenomenon can be attributed to the amplifying weight of overlaying soil strata that escalates with depth, leading to organic matter depletion. Such pressures from above layers compact the layers beneath, culminating in reduced pore spaces and an augmented BD [61]. This trend is conspicuously marked in soils that are either compacted or structurally compromised. Over-reliance on mechanical interventions can momentarily expand pore space, reducing BD at the plow layer, but concurrently raising the risk of a hard pan forming just beneath. Interestingly, ZT plots, due to minimal disruptions, typically showcased higher BD values across diverse depths, an observation echoed by Ref. [62]. As the experiment concluded, significant variations in BD were largely confined to the superficial layer (0–15 cm). Beneath this stratum, differences in BD between MT and CT practices were statistically negligible [63]. The consistency of ZT in delivering elevated BD across depths is rooted in its non-invasive approach. Yet, from a long-term perspective, ZT showcases fewer compaction concerns compared to alternative tilling techniques [64]. Moreover, ZT tends to slow down organic decomposition at shallower strata, fostering soil carbon accumulation, an attribute that could spell long-term benefits for soil health [65]. While mulching undoubtedly influences a gamut of soil traits, such as moisture retention, SOC, and temperature equilibrium, its bearing on BD remains comparatively muted [29]. This isn't to undermine mulching's role in soil dynamics; its impact on BD could be modulated by a myriad of factors, including initial soil health, mulch type and depth, and the mulch's application duration [66]. Of the mulching methodologies employed, live mulching was distinctly superior in the uppermost soil layers. The continuous introduction of organic matter via the symbiotic plant's root activity and its eventual decomposition could elucidate its marked efficacy in the 0–15 cm range. In contrast, strategies like paddy straw and leaf litter mulching involve periodic organic matter deposition that decays gradually.

The ZT practices recorded higher SOC sequestration rates and a net increase in SOC content compared to MT and CT. This suggests that ZT is more effective in preserving and increasing SOC by employing physical protection [67,68]. Minimal soil disturbance, which permits higher protection of carbon from microbial attack in the aggregates, reduces the organic matter mineralization rate and accounts for the significant increase in carbon stocks in superficial layers in ZT plots [69,70]. On the other hand, CT involves mechanical soil disturbance through intensive tillage which can accelerate the decomposition of organic matter and increase soil erosion, reducing SOC levels over time [71]. Following this pattern, it was found that using CT in a rice-sweet corn cropping system resulted in a net loss of SOC. To some extent, MT is preferable to CT practices because it slows the rate at which SOC content decreases over time and after cropping [72]. However, unlike ZT, it is not effective in significantly boosting SOC levels. Similar findings were also reported by Refs. [73,74]. Similarly, mulching regimes had varying effects on soil carbon sequestration and SOC content. In general, the effectiveness of mulching in adding SOC depends on various factors, including the type and quality of the organic material used, decomposition rates, climate, soil type, and management practices [62,75]. In the current experiment, it was observed that live mulching and leaf litter mulching were far superior to paddy straw mulching in terms of the amount of carbon added to the soil and the rate at which carbon was sequestered. Mulching with live mulch can provide a steady supply of organic material via their root and leaf exudates and decaying or living above-ground biomass. A steady supply of carbon in the form of organic matter addition aids in SOC build-up [76]. Since leaf litter from trees and shrubs has a faster decomposition rate than other mulch materials like straw, this may contribute to the observed result. Due to their increased nutritional content, lower lignin levels, and lower C–N ratio, leaf litter mulch is likely to decompose more quickly in the presence of soil microbes than carbon-rich paddy straw mulches. In contrast, live mulch can stay alive during the entire crop growth season [77]. A similar beneficial report of leaf litter mulch against straw and saw-dust mulch has been reported by Ref. [78] in the northern Iranian province. Intensive tillage practices have not only depleted SOC in rice-based cropping systems but also resulted in a decline in crop yields over the years [79]. In the current experiment, a significant decrease in both the crop and system yield was observed in ZT as compared to CT practices. Yield reductions in the ZT system were also recorded by Ref. [80]. This might be due long-term adaption period of ZT systems. When switching to ZT, the soil and crops need time to react to the new management system. Short-term negative impacts on yield can occur during this transition phase, but they are typically expected to be temporary [81,82]. The absence of soil disturbance in ZT can impact the population dynamics of certain weeds, pests, and diseases, which may be another reason for yield losses [83,84]. However, implementing appropriate integrated pest and disease management strategies specific to ZT can help mitigate the potential yield losses. It was also observed that the reductions in crop and system yield in the case of MT were minor. Similar findings were also reported by Refs. [85,86]. This similarity in yield patterns indicates that MT can be a viable alternative to CT without compromising overall productivity in a shorter transition period before switching to ZT.

Organic mulching regimes, especially live and leaf litter mulching, have been associated with augmented crop productivity. The underlying reasons encompass a multitude of benefits conferred by mulching: it ensures consistent water availability to plants by curbing excessive evaporation, suppresses weed proliferation, provides thermal equilibrium, and amplifies nutrient cycling following decomposition [[87], [88], [89]]. Particularly, live and leaf litter mulching outpaces paddy straw mulching in enhancing crop yields, largely attributable to their superior nutrient recycling capabilities. This enhanced nutrient cycling bolsters soil fertility, securing an uninterrupted nutrient flow to plants [90]. Furthermore, live mulching, especially when done using legumes, introduces additional nitrogen to the soil and fosters a thriving rhizospheric microbial community, adding another layer of benefit for crops. Conversely, paddy straw mulching, while still beneficial, might be less adept at obstructing weed emergence and spread compared to its counterparts [91].

Regarding cost considerations, it was observed that the costs incurred in ZT and MT were lower. In CT, use of expensive and/or heavy machinery such as ploughs, harrows, and cultivators at multiple times causes increased hiring, labour and fuel costs. In contrast, no-till drills and seeders are often smaller, easier, and cheaper to use in ZT systems, several unnecessary tillage operations are completely omitted. By switching to ZT or MT one can save fuel, maintenance, and labour costs [92]. The B: C ratio is more important than the net return when determining the economic efficiency of a cropping system. Even though CT practices led to a higher NR, its inferior B: C ratio compared to ZT and MT reflects its poor economic efficiency. In mulching treatments, live mulching has been shown to be the most cost-effective method, followed by mulching with leaf litter. This could result from the fact that they are frequently available on farms or in proximity, making them an affordable choice. Mulching with living material or leaf litter may be less labour-intensive than mulching with paddy straw [93].

Tillage regimes have significantly influenced system energetics and environmental footprints of the rice-sweet corn cropping system in the current experiment. Tillage activities need fuel-powered machineries like tractors and ploughs, increasing input energy [94]. Excessive tilling, for instance, could damage the soil's structure and reduce its ability to hold nutrients and water, necessitating greater energy inputs for fertilization and irrigation [95,96]. Maximum energy output was recorded in CT. Similar patterns were seen between output energy and net energy. Considering that energy output alone may not provide a complete picture of the sustainability and environmental impact, consideration of other sustainability parameters is pivotal. Factors such as soil health, water usage, GHG emissions, and long-term sustainability should be evaluated to make more comprehensive conclusions about the benefits and trade-offs of different tillage methods. Taking those into account, MT emerges as the superior tillage approach since it maintains soil health, is cost-effective, and yields outcomes on par with those of CT practices in the short term while after stabilization ZT can potentially improve the system's performance [26,39]. This experiment showed that live mulching had the highest net energy and energy output. The active growth and photosynthesis of the live mulch plants may account for the increased energy output observed in live mulching [41]. Mulching with leaf litter can result in faster decomposition of mulch materials, which enriches the soil with nutrients and stimulates microbial activity [97]. However, paddy straw may have a lower energy production because of its sluggish decomposition rate and the residue's poor nutritional content [45].

In the examined system, Conventional Tilling (CT) evidenced diminished energy productivity and profitability due to its intensive energy inputs. In stark contrast, Zero Tillage (ZT), under its minimalistic energy consumption during land preparation and sowing, emerged as the most energy-efficient approach—a finding consistent with [92]. Moreover, Minimum Tillage (MT) also showcased significantly superior energy productivity and profitability in comparison to CT. However, when examining mulching strategies, the study revealed that paddy straw mulching lagged in both energy productivity and profitability compared to other methods such as live mulching, leaf litter mulching, or even the absence of mulching. This can be attributed to the elevated energy inputs demanded by paddy straw mulching, a nuance highlighted by Ref. [45]. Contributing factors might encompass the labor and energy-intensive processes of straw collection, transportation, and distribution across fields, coupled with the inherent challenges posed by straw decomposition. Among all mulching variants explored, live mulching emerged as the most energy-productive and profitable strategy.

Any farming system's CF and contribution to global warming heavily relies on its tillage and soil cover management practices. Intensive tillage practices tend to increase GHG emissions by accelerating the decomposition of soil organic matter and releasing carbon dioxide, and nitrous oxides into the atmosphere. Soil organic matter content and carbon sequestration can be increased by continuously adding organic matter, like crop residues, leaf litter, or living plants as well as conservation of the added carbon through minimal soil disturbance. Our findings suggest that CT practices increase the CF of cultivating rice and sweet corn. The CF can be reduced by as much as three-quarters use ZT approaches and by as much as one-quarter using MT practices. Consistent with the previous trends, the GWP was highest when CT was adopted, but it could be significantly reduced when ZT and MT methods were implemented [98]. In the case of mulching, one interesting change in the pattern was observed. In general, mulching practices can contribute to increased GWP by increasing microbial activity, breaking down the organic matter, and releasing CO_2_ as a by-product of respiration. Thus, compared to no mulching, the GWP was more remarkable for both leaf litter and paddy straw mulch. However, live mulch can act as a carbon-capturing mechanism using biomass generation in a short time and finally adding that carbon to the soil in the long run.

An overall comparison among the similar studies conducted reveled that a wide variation in input, output, net energy, and carbon footprint in case of rice-maize production systems. This was since agricultural input and outputs are shaped by local soil-plant-climatic-managemental landscapes. Thus, such studies cannot be generalized and must be seen differently under different agroclimatic, soil, crop and managemental scenarios.

## 5 Conclusion

The present study found that ZT resulted in consistently higher bulk densities throughout different soil depths due to initial no-till compaction, whereas MT and CT had similar bulk densities. However, in the initial years also ZT plots had significantly higher SOC and showed a higher sequestration rate. A net loss of SOC was recorded with CT while MT maintained SOC levels. Live mulching had the greatest positive impact on soil carbon storage, sequestration rate, and system performance. Contrary to the beneficial impact on soil and climate, ZT resulted in reduced system and componential yield than both MT and CT. whereas; MT and CT yields were comparable. Though GR was highest for CT, ZT resulted in a significant decrease in the CoC and scored the highest B: C ratio indicating good economic efficiency. CT also had been recorded with the highest energy requiring treatment whereas ZT saved a lot of energy and resulted in a significant reduction of CFs and GWPs. In the current study, a mixed response was observed in terms of better performance of ZT in soil, energy, and environmental parameters and poor performance in terms of yield and economic benefits. MT nearly scored in midway between ZT and CT. Poor system performance and yield penalties in ZT in the initial years may cause a financial burden on farmers despite promising future opportunities and sustainable returns over time. Keeping this in consideration, the current study advocates the use of MT in the initial years and a gradual shift to ZT can offer benefits of conservation tillage and returns of conventional ones. Among the mulching regimes, live mulching with cowpea was found most suitable soil cover management with all the tillage regimes. However, it's essential to consider site-specific factors and long-term effects when choosing the appropriate tillage and mulching practices for a specific cropping system.

## Data availability statement

The current manuscript has no data associated that has been deposited into a publicly available repository. Data may be made available on reasonable request.

## CRediT authorship contribution statement

**Arindam Kundu:** Investigation, Conceptualization. **Champak Kumar Kundu:** Investigation, Formal analysis, Conceptualization. **Prithwiraj Dey:** Writing – original draft, Validation, Methodology, Formal analysis, Data curation. **Soham Rana:** Writing – original draft, Software, Formal analysis. **Jhumur Majumder:** Software, Formal analysis, Data curation. **Anurag Bera:** Formal analysis, Data curation. **Bappa Paramanik:** Formal analysis, Data curation. **Partha Sarathi Patra:** Formal analysis, Data curation. **Md Galal Uddin:** Writing – review & editing. **Mohamed Abioui:** Writing – review & editing. **Anuj Saraswat:** Writing – review & editing.

## Declaration of generative AI and AI-assisted technologies in the writing process

During the preparation of this work, the authors did NOT use any of the generative AI and AI-assisted technologies in the writing process. The authors reviewed and edited the content as needed and take full responsibility for the content of the publication.

## Declaration of competing interest

The authors declare that they have no known competing financial interests or personal relationships that could have appeared to influence the work reported in this paper.
